# Effect of Soy Protein Isolate on Textural Properties, Cooking Properties and Flavor of Whole-Grain Flat Rice Noodles

**DOI:** 10.3390/foods10051085

**Published:** 2021-05-14

**Authors:** Zhongfu Cao, Yang Liu, Hong Zhu, Yisi Li, Qian Xiao, Cuiping Yi

**Affiliations:** 1 School of Chemistry and Food Engineering, Changsha University of Science and Technology, Changsha 410114, China; cuddie@163.com (Z.C.); liuyangcsust@163.com (Y.L.); 15111230149@163.com (H.Z.); liyisi1995@163.com (Y.L.); 2 School of Food Science and Technology, Hunan Agricultural University, Changsha 410128, China; qianxiao@hunau.edu.cn

**Keywords:** whole-grain product, flat rice noodles, soy protein isolate, quality, flavor

## Abstract

To investigate the effect of soy protein isolate on the quality of whole-grain flat rice noodles, the texture as well as the cooking properties and flavor of flat rice noodles, whole-grain flat rice noodles and whole-grain flat rice noodles with soy protein isolate were investigated. Among the three tested rice noodles, whole-grain flat rice noodles with soy protein isolate showed the highest cohesiveness, adhesiveness, resilience, and springiness. Compared to the flat rice noodles and whole-grain flat rice noodles, whole-grain flat rice noodles with soy protein isolate increased their moisture content and water absorption, whereas the opposite trend was observed for their cooking loss. The electronic nose analysis showed stronger response values at W5S, W1W, and W2W. Solid phase micro extraction and gas chromatography–mass spectrometry results showed that aldehydes are the main volatile compounds in whole-grain flat rice noodles and whole-grain flat rice noodles with soy protein isolate. Moreover, seven more volatile compounds were detected in whole-grain flat rice noodles with soy protein isolate compared to flat rice noodles and whole-grain flat rice noodles. The whole-grain flat noodles with the addition of SPI are more sensory acceptable. Thus, soy protein isolate, as a natural and safe additive, could be used to improve the quality and enrich the flavor of whole-grain flat rice noodles.

## 1. Introduction

Flat rice noodles (FRN), produced from Indica polished rice, are a very popular staple food in Southeast Asian countries. Brown rice is a de-hulled grain consisting of bran, embryo, and endosperm layers, which is rich in dietary fibers, phenolics, vitamins, and minerals [1]. According to epidemiological studies, the increased consumption of whole grains and/or whole grain products has been associated with reduced risk of chronic diseases, including cardiovascular disease, type II diabetes, obesity, and cancer [2,3,4]. Therefore, the addition of rice bran into refined flat rice noodles to produce whole-grain flat rice noodles is a suitable way to improve the nutritional quality of flat rice noodles.

Previous studies have reported that flat rice noodles made from whole-grain rice showed poor texture properties and cooking qualities, because they have a rough surface and are easy to break [5]. Many studies have focused on improving the quality of whole-grain rice noodles involving different approaches such as slight milling, cellulase enzymatic treatment [6], and heat-moisture treatment [7]. These methods are relatively expensive. An appropriate amount of natural additives is another way to improve the edible quality, textural properties, and nutritional quality of rice noodles. Previous studies have demonstrated that the appropriate addition of wheat, pinto bean, polished rice, and corn meal can improve the quality of rice noodles [8]. In recent years, soy protein isolate (SPI) have received special attention due to their availability, well balanced amino-acid composition, health benefits, and ability to improve texture properties and retain water in food products [9]. SPI is a complex mixture of proteins, mostly globulins with numerous applications in meat analogues products [10], gluten-free foods, and cereal-based products [11]. SPI addition can improve the nutritional qualities, digestibility properties, and other characteristics of banana pasta [12] and rice dough [13]. At present, there is no report about the effect of SPI on the quality of flat rice noodles. Our preliminary experimental results showed that with increasing SPI concentration from 0 to 9%, the breaking distance of whole-grain flat rice noodles increased, whereas the opposite trend has been observed with further increase of SPI concentration. Therefore, 9% SPI was used in this experiment.

Flavor is an important factor that affects the quality of rice products and their popularity [14]. At present, there is no research on the flavor compounds of flat rice noodles, and the change of flavor after SPI addition is still unclear. The electronic nose (E-nose) is an efficient and simple tool for food flavor assessment. It usually consists of many sensitive and high detection speed electronic chemical sensors [15]. Solid phase micro extraction and gas chromatography–mass spectrometry (SPME/GC-MS) has gained popularity for qualitative and quantitative analyses of volatile compounds [16]. The advantages of this method are convenience, good reproducibility, high speed, low sample requirements, and simple operation. However, neither SPME/GC-MS nor E-nose technology have been reported to be used in the flavor analyses of flat rice noodles or SPI-added whole-grain flat rice noodles.

For the improvement and development of whole grain brown flat rice noodles, the objectives of this study were to investigate the effects of SPI on the texture properties, cooking property, and flavor of whole-grain flat rice noodles.

## 2. Materials and Methods

### 2.1. Materials

A brown rice (*Oryza sativa L.*) cultivar (*indica* rice, Xingyue) was used in this study. It was grown in Ningxiang (Hunan, China) during the 2017 growing season and harvested in July. After being dehulled, brown rice grains were ground into polished rice and rice bran by a milling machine (THU35C, Zuozhu Machinery Co. Ltd., Suzhou, China). Every 100 g brown rice contains 7 g rice bran. They were stored in sealed polyethylene containers at −4 °C. Soy protein isolate (Shansong Biological Products Co., Ltd. Linyi, China) contains 96% protein.

### 2.2. Preparation of Flat Rice Noodles

Flat rice noodles (FRN): The polished rice were soaked in excess tap water at 25 °C for 4 h, and they were ground into rice slurry with a refiner (SY-12, Zhe Jiang Shark Food Machingery Co., Ltd., Lishui, Zhejiang, China). The slurry was evenly poured on a stainless steel stray and steamed at 100 °C for 6 min. The rice noodle sheet was removed from the tray and cut into 20 cm × 1.5 cm strands.

Whole-grain flat rice noodles (WFRN): The polished rice was soaked in excess tap water at 25 °C for 4 h, and they were ground into rice slurry with a refiner. Then, 100.00 g rice slurry was mixed with 7.00 g rice bran. The remaining steps are the same as FRN.

Whole-grain flat rice noodles with soy protein isolate (WFRN-SPI): The polished rice was soaked in excess tap water at 25 °C for 4 h, and they were ground into rice slurry with a refiner. Then, 100.00 g rice slurry was mixed with 7.00 g rice bran and 9.00 g soy protein isolate; the ratio was optimized by our laboratory. The remaining steps are the same as FRN.

### 2.3. Textural Properties

The texture properties of three flat rice noodles were analyzed using a TA-XT 2i/5 Texture Analyzer (Stable Micro System Ltd., Godalming, England) as previously reported with some modifications [17]. Three types of flat rice noodles with uniform shapes were placed under the P/36R probe (36 mm diameter cylindrical probe). The parameters were set as follows, pre-test speed 2 mm/s, test speed 1 mm/s, return speed 5 mm/s, residence time 5 s, 50% compression ratio, 0.05 N triggering force, and a 3 s compression interval. Each sample was measured 10 times.

### 2.4. Cooking Properties

The cooking loss of the flat rice noodles was analyzed according to AACC procedure 66–50 [18]. After being boiled in 150 mL water for 1 min, 10 g flat rice noodles were separated from the cooking water and then weighed. The cooking water was dried to a constant weight in an oven at 105 °C. The cooking loss was indicated by the percentage of solid loss during cooking. Rice noodle samples were prepared in triplicate. The water absorption was analyzed according to a previous study with some modifications [19], and it was given by the amount of weight gain by the flat rice noodles after cooking. The increase in weight of the rice noodles on cooking was determined as the water uptake ratio as a percentage. The moisture content was determined by direct drying method.

### 2.5. Scanning Electron Microscope (SEM)

The samples were then freeze-dried at −38 °C, 10 Pa, for 24 h and put on a circular specimen stub and attached. The surface was sprayed with gold, and the samples were observed using a Quanta FEG 250 SEM (FEI Co., Hillsboro, OR, USA). The external microstructure of flat rice noodles was observed 2000 times. The detector was ETD. All images were obtained at the accelerating voltage of 15 kV.

### 2.6. Electronic Nose Analysis

The measurement parameters were according to the procedures described by Chen [20] with some modifications. Seven grams of flat rice noodles were placed in 150 mL headspace bottles and stored at 4 °C for 1 h. Subsequently, an equilibration process was performed at 25 °C for 30 min before electronic nose analysis (PEN3, Airsense, Germany). The experiments were carried out fivefold.

### 2.7. SPME/GC-MS Analysis

The volatile properties of flat rice noodle samples were analyzed and identified according to our previous study [21].

### 2.8. Sensory Evaluation

In order to test the acceptability of three kinds of flat rice noodles, a sensory evaluation was carried out according to Cocci [22] with some modifications. A group of 30 trained panelists (half was male and half was female) was invited to evaluate the sensory attributes. Appearance, flavor, taste, hardness, resilience, smoothness, and viscosity, accounted for 15, 15, 15, 15, 15, 15 and 10 points of the total score respectively. “0” means “Extremely Dislike” on this indicator, and full score (10 or 15) means “Extremely Like” on this indicator.

### 2.9. Statistical Analysis

Experiments were performed in triplicate, and the average value was expressed as the mean ± standard deviation (SD) unless mentioned otherwise, which was calculated with Origin 9.1 software (OriginLab Co., Northampton, MA, USA). Experimental data were statistically analyzed by one-way analysis of variance (ANOVA) in SPSS 19.0 software (IBM Co., Armonk, NY, USA), and *p* < 0.05 was considered statistically significant.

## 3. Results and Discussion

### 3.1. Textural Properties

The cohesiveness, adhesiveness, resilience, and springiness values of flat rice noodles are presented in Table 1. The results showed that the cohesiveness, adhesiveness, resilience, and springiness values of WFRN were significantly lower than that of FRN (*p* < 0.05), while no significant difference was observed for their hardness and chewiness values. Textural properties of noodle are mainly affected by the matrix structural network of starches, glutens, other proteins, fibers, and other additional ingredients. The addition of rice bran with high fiber content would weaken the formation of hydrogen bonds within the noodle structure network, which leads to deterioration of textural properties [23]. Besides, the reduction of amylose content may result in a decrease in gel properties and textural properties of flat rice noodles [24].

The cohesiveness and springiness of WFRN-SPI and FRN had no significant difference (*p* > 0.05), while the hardness of WFRN-SPI was significantly lower than WFRN. Compared to WFRN, FRN showed higher resilience and springiness values; this may be because rice protein and starch formed a network structure. However, the fiber in rice bran hindered the formation of hydrogen bonds within the WFRN. For WFRN-SPI, a porous honeycomb network structure appeared on flat rice noodles; this may be because soy protein isolate interacts with rice starch. Thus, similar textural properties of FRN and WFRN-SPI have been observed. The hardness and chewiness of WFRN-SPI were significantly lower than that of WFRN and FRN, due to the higher water holding capacity of SPI [25].

### 3.2. Cooking Properties

**Figure 1:**
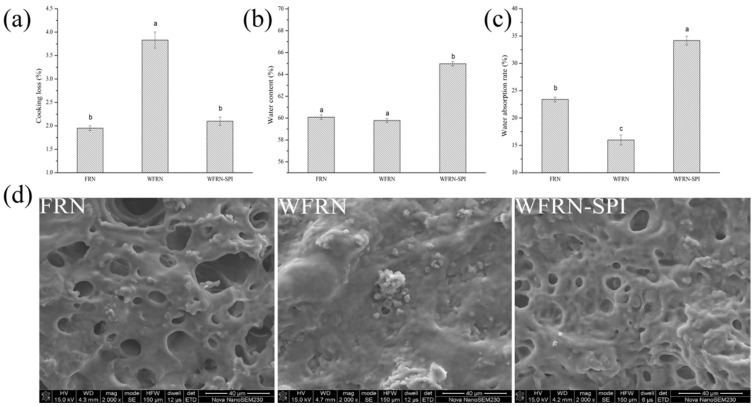
Cooking properties and scanning electron microscopy (SEM) images of flat rice noodles. (**a**–**d**) represent cooking loss, moisture content, water absorption, and SEM images respectively; FRN, WFRN, and WFRN-SPI represent the flat rice noodles, whole-grain flat rice noodles, and whole-grain flat rice noodles with soy protein isolate, respectively. Different small letter superscripts in the same histogram are significantly different at *p* < 0.05.

Cooking loss is an important index to reflect the cooking quality of flat rice noodles, because the loss on cooking shows resistance to disintegration during cooking. Figure 1a–c showed the cooking properties of the flat rice noodles. The cooking loss of FRN, WFRN and WFRN-SPI was 1.95%, 3.83% and 2.11% respectively. Similar to previous reports [26], the addition of wheat bran to noodles significantly increased the cooking loss of noodles. This could be attributed to the destruction of the protein-starch network by the high fiber content of bran, which allowed more of the gelatinized starch to leach from the noodles during cooking [27]. However, no significant difference was observed between WFRN-SPI and FRN. The cross-linking among protein molecules can create an extensive network that acts as a barrier against water penetration [28].

Water absorption and the moisture content are correlated with the cooking properties of starch-based noodles. The water absorption of FRN, WFRN, and WFRN-SPI was 23.41%, 15.99%, and 34.17% respectively. The water absorption of WFRN was significantly lower than that of FRN; this result was in agreement with reports that insoluble dietary fiber decreased the water absorption of noodle samples, due to the binding of water competes with starch [29]. However, the water absorption of WFRN-SPI was significantly higher than that of FRN and WFRN. The main reason for water absorption of flat rice noodles is starch gelatinization, followed by protein hydration. SPI has good water absorption, water retention, and expansion, due to many polar groups on the peptide chain skeleton of SPI. It has also been reported that the water absorption of noodles decreased when the SPI content was low, because the gluten network was strengthened [30].

The moisture content of FRN, WFRN, and WFRN-SPI was 60.09%, 59.78%, and 64.98% respectively. The moisture content of WFRN-SPI was significantly higher than that of FRN and WFRN, which was mainly attributed to the water absorption of SPI during steam-cooking.

### 3.3. SEM

The SEM of FRN, WFRN, and WFRN-SPI is presented in Figure 1d. There was a network structure on the surface of FRN, but this phenomenon was not observed in WFRN. However, the addition of SPI led to the network structure reappearing in WFRN-SPI. Similar phenomena have been reported; after SPI was added to gluten-free rice spaghetti, the SEM showed the more porosity at the surface, and the fluorescent micrograph showed that the proteins were arranged neatly in starch [31]. The mixture of rice flour and SPI has more dense and compact structure, which led to better cooking and texture quality [32]. SPI has the capacity to form a three-dimensional skeleton and make the microstructure compact [33].

### 3.4. Electronic Nose Analysis

**Figure 2:**
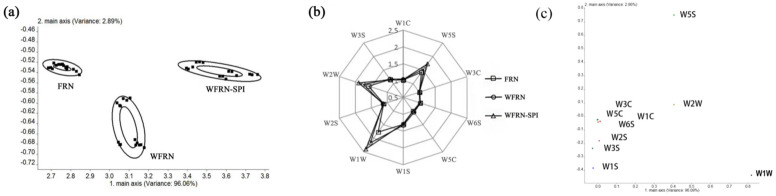
Electronic nose analysis of flat rice noodles (**a**–**c**) represents principal component analysis (PCA) results, response value of 10 sensors, and loading analysis, respectively; FRN, WFRN, and WFRN-SPI represent the flat rice noodles, flat rice noodles, whole-grain flat rice noodles, and whole-grain flat rice noodles with soy protein isolate, respectively.

The results of electronic nose analysis of flat rice noodles are shown in Figure 2. Principal component analysis (PCA) showed that the cumulative variance of the first principal component (PC1) was 98.06%; the second principal component (PC2) was 2.89%; and PC1 and PC2 accounted for 99.74% of the total variance. PCA images of the three flat rice noodles samples did not overlap each other, indicating that they are different odors and can be distinguished by electronic nose. With the addition of rice bran and SPI, the distribution of volatile compounds in flat rice noodles changed along the direction of PC1.

Similar radar plots have been observed for the flavor of the three kinds of flat rice noodles. They all had obvious response values at W5S (sensitive to NO_x_), W1W (sensitive to sulfide and sulfur-containing organic compounds) and W2W (sensitive to aromatic compounds and organic sulfides). The result was consistent with the analysis of flavor compounds in rice noodles [34]. WFRN-SPI had the strongest response values of W5S, W1W, and W2W, followed by WFRN and FRN. This finding suggested that the flavor of nitrogen oxides, sulfide, sulfur-containing organic compounds, aromatic compounds, and organic sulfur compounds was enhanced with addition of rice bran and SPI. Loading analysis of volatile compounds from flat rice noodles showed that the contribution rates of the first and second principal components in loading analysis were 96.06% and 2.89% respectively, and the total contribution rate was 98.95%, indicating that the analysis results contained the main sample information. The sensors W5S, W1W, and W2W made the biggest contribution to the first principal component, while the sensors W5S, W2W, and W3C (sensitive to ammonia and aromatic compounds) made the biggest contribution to the second principal component. The sensors W5S, W1W, and W2W are farthest from the origin, so they played a key role in distinguishing the volatile compounds of three flat rice noodles.

### 3.5. Volatile Compound Analysis

**Figure 3:**
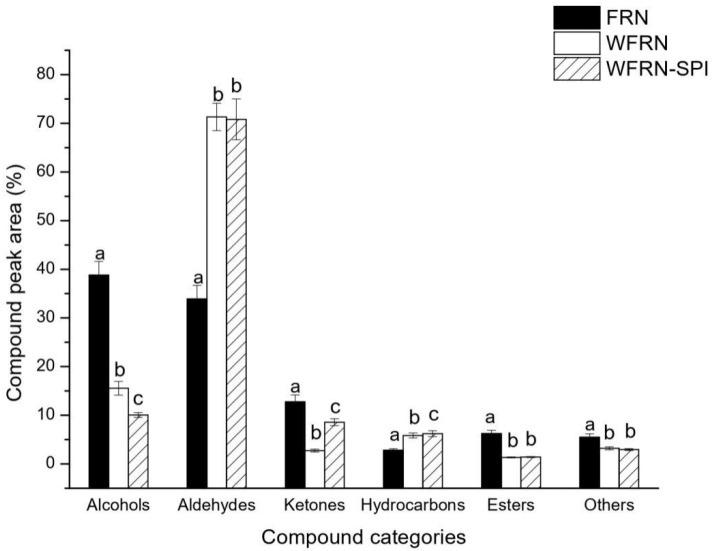
Category distributions of volatile compounds from flat rice noodles. Compound peak area, the percentage of the area of each category to total area of identified compounds; FRN, WFRN, and WFRN-SPI represent the flat rice noodles, whole-grain flat rice noodles, and whole-grain flat rice noodles with soy protein isolate, respectively. Different small letter superscripts in the same group of histograms are significantly different at *p* < 0.05.

The volatile components of flat rice noodles are presented in Table 2. Our measurements of volatile components in FRN, WFRN, and WFRN-SPI showed 24, 34, and 41 components respectively. The types of volatile compounds were enhanced by the addition of rice bran and SPI. The category distributions of volatile compounds from flat rice noodles is shown in Figure 3.

Alcohols were the dominant volatile compounds in FRN, WFRN, and WFRN-SPI, and their contents were 38.80%, 15.55%, and 10.05% respectively. The highest relative content of 2-Undecen-1-ol, (2E)- was in FRN (36.40%). 1-octen-3-ol was the most important alcohol contributing flavor in rice and rice product, because of the high threshold and low content of other alcohols [35]. 1-octen-3-ol, mushroom flavor, was detected only in WFRN and WFRN-SPI, and was enhanced with the addition of SPI. Table 2 demonstrated that FRN had the highest relative content of alcohols, but WFRN and WFRN-SPI had more kinds of alcohols, especially WFRN-SPI.

Aldehydes were the dominant volatile compounds in WFRN and WFRN-SPI, accounting for 71.31% and 70.80% respectively. The content of aldehydes in FRN was significantly lower than the other two type of flat rice noodle, due to the relatively high content of fatty acids in rice bran. However, aldehydes and alcohols identified by SPME are mainly the products of fatty acid degradation and oxidation [36]. Thirteen types of aldehydes were detected in WFRN-SPI, which was more than that of flat rice noodles. 2,4-decadienal, 4-nonenal, (4E)-, and dodecyl aldehyde were only detected in WFRN-SPI. Octanal was detected in all three kinds of flat rice noodles, but hexanal was only detected in WFRN and WFRN-SPI. Octanal and hexanal are degradation products of oleic acid, and their odor descriptor is green [37]. 1-nonanal content of FRN, WFRN, and WFRN-SPI was 23.87%, 31.18%, and 20.73% respectively, and its odor descriptor was green, citrusy, and soapy, respectively [38].

Ketones, fruit, and floral aroma are important aroma components in FRN and WFRN-SPI, such as 2-heptanone and (1R)-trans-p-menthan-3-one; similar results have been reported in fragrant rice [39]. Esters are among the unsaturated fatty acids degradation products that appear at the late stages of the oxidation process [40]. Few esters were detected in all flat rice noodles. The hydrocarbons identified included alkanes, terpenes, and aromatic compounds, and they are known as the secondary products of unsaturated fatty acids oxidation. A small amount of indole, showing tar smell [41], was detected in FRN and WFRN, while indole was not detected in WFRN-SPI.

Although similar sensors response characteristics of three types of flat rice noodles have been determined by electronic nose analysis (Figure 2b), SPME/GC-MS analysis showed that they had different variation trends in flavor compounds (Figure 3). This could explain that the main odor of flat rice noodles consisted of a number of volatile compounds [42].

### 3.6. Sensory Evaluation

**Figure 4:**
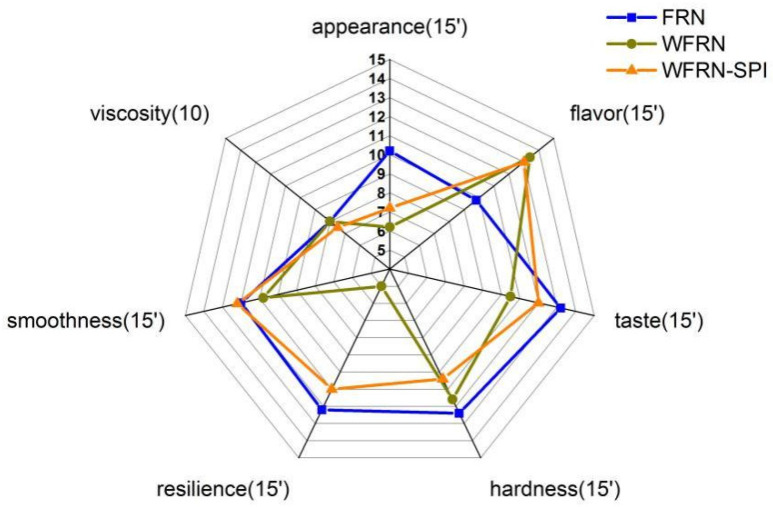
Sensory evaluation of flat rice noodles. FRN, WFRN, and WFRN-SPI represent the flat rice noodles, whole-grain flat rice noodles, and whole-grain flat rice noodles with soy protein isolate, respectively.

Results of flat rice noodles sensory acceptability are shown in Figure 4. The scores in the figure indicated the preference of consumers for each attribute. FRN had the highest appearance score; this may be because consumers still preferred traditional white flat rice noodles. There were clear differences in hardness, resilience, and smoothness of samples, which was consistent with the texture results above. WFRN and WFRN-SPI had higher scores in flavor, and this result was consistent with the electronic-nose and volatile compounds’ results above. In summary, the global sensory scores of WFRN-SPI were similar with FRN, and the quality of WFRN was not good enough to meet the basic needs of consumers. The sensory evaluation indicated that the quality of WFRN with SPI addition met the basic demands of consumers.

## 4. Conclusions

This study demonstrated that the addition of rice bran reduced the resilience and increased the cooking loss of flat rice noodles by destroying the network structure. The addition of SPI improved the texture properties and cooking properties of whole-grain flat rice noodles; this may be because of the formation of dense soy protein network structures. SPME/GC-MS analysis showed that adding rice bran and SPI both enriched the flavor of flat rice noodles. The main volatile compounds changed from alcohols to aldehydes. The whole-grain flat noodles with the addition of SPI are more sensory acceptable. Adding 9% SPI is a simple and economical way to produce whole-grain flat rice noodles with idea quality. These results are beneficial to the comprehensive utilization of whole-grain resources and lay the foundation for whole-grain products deep processing technology.

## Figures and Tables

**Table 1:** Textural properties of flat rice noodles.

	Cohesiveness	Adhesiveness (N·s)	Resilience	Springiness	Hardness (N)	Chewiness(N)
FRN	0.76 ± 0.03 ^b^	−2.23 ± 0.55 ^b^	0.507 ± 0.01 ^b^	0.96 ± 0.01 ^b^	51.30 ± 4.85 ^a^	37.37 ± 3.26 ^a^
WBRN	0.71 ± 0.03 ^a^	−1.57 ± 0.23 ^a^	0.390 ± 0.01 ^a^	0.91 ± 0.01 ^a^	47.93 ± 6.11 ^a^	32.17 ± 5.08 ^ab^
WBRN -SPI	0.74 ± 0.04 ^b^	−1.87 ± 0.24 ^a^	0.437 ± 0.01 ^ab^	0.95 ± 0.01 ^b^	35.42 ± 3.94 ^b^	24.52 ± 3.17 ^b^

FRN, WFRN, and WFRN-SPI represent the flat rice noodles, whole-grain flat rice noodles, and whole-grain flat rice noodles with soy protein isolate, respectively; Means values ± standard deviation, n = 10. Different superscript letters in the same column are significantly different at *p* < 0.05.

**Table 2:** Volatile compound analysis of flat rice noodles.

Compounds	Relative Content (%)		
	FRN	WFRN	WFRN-SPI
**Alcohols**			
2-Undecen-1-ol, (2E)-	36.40 ^a^	0.49 ^b^	-
Dihydroterpineol	2.02 ^b^	3.94 ^a^	3.73 ^a^
isomenthol	0.18	-	-
1-Pentanol	-	6.37 ^a^	3.27 ^b^
1-Octen-3-ol	-	0.44 ^b^	1.74 ^a^
2-Decyn-1-ol	-	1.95	-
3,5-Octadien-2-ol	-	0.09 ^b^	0.43 ^a^
1-Octanol	-	2.05 ^a^	0.18 ^b^
(2Z)-2-Octene-1-ol	-	-	0.13
2-Pentadecyn-1-ol	-	-	0.24
Linalool	0.20 ^a^	0.22^a^	-
2-Decen-1-ol, (2E)-	-	-	0.15
alpha-Terpineol	-	-	0.18
**Aldehydes**			
1-Nonanal	23.87 ^b^	31.18 ^a^	20.73 ^c^
Hexanal	-	25.26 ^b^	30.96 ^a^
2-Nonenal, (2E)-	1.25^b^	1.62 ^a^	1.27 ^b^
Decanal	4.67 ^a^	2.90 ^b^	2.85 ^b^
Octanal	3.83 ^c^	5.80 ^a^	4.24 ^b^
Heptanal	-	2.72 ^b^	3.63 ^a^
Dodecanal	0.28 ^a^	0.30 ^a^	0.31 ^a^
(E)-2-Octenal	-	1.04 ^b^	2.69 ^a^
2,4-nonadienal	-	0.22 ^a^	0.23 ^a^
trans-2-Decenal	-	0.27 ^b^	0.51 ^a^
2,4-decadienal	-	-	2.81
4-Nonenal, (4E)-	-	-	0.18
Dodecyl aldehyde	-	-	0.39
**Ketones**			
(1R)-trans-p-menthan-3-one	9.04 ^a^	1.07 ^c^	2.58 ^b^
Geranylacetone	1.47 ^a^	0.98 ^b^	0.99 ^b^
2-Dodecanone	-	0.43 ^a^	0.31 ^b^
2-Heptanone	-	-	2.96
3,5-Octadien-2-one,(3E,5E)-	-	-	1.12
1-(3-Butyloxiranyl)ethanone	-	-	0.44
4-Nonenal, (4E)-	-	-	0.17
6-Methyl-5-hepten-2-one	1.75	-	-
2-Nonanone	-	0.26	-
5-methyl-2-(1-methylethylidene)-Cyclohexanone	0.50	-	-
**Hydrocarbons**			
Tridecane	1.29 ^b^	1.62 ^a^	1.04 ^c^
Cyclopropane, pentyl-	-	2.44 ^a^	1.25 ^b^
Dodecane, 4,6-dimethyl-	-	0.57 ^a^	0.45 ^b^
Decane,6-ethyl-2-methyl-	-	0.33 ^b^	0.62 ^a^
Undecane, 3,5-dimethyl-	0.8	-	-
Heptadecane, 2,6-dimethyl-	0.74	-	-
Heptadecane,2,6,10,14-tetramethyl-	-	0.88 ^b^	2.31 ^a^
2,6,8-Trimethyldecane	-	-	0.55
**Esters**			
Phenacyl thiocyanate	2.08 ^a^	0.12 ^b^	0.12 ^b^
Formic acid, heptylester	2.54 ^a^	1.23 ^b^	1.29 ^b^
Arecaidine methyl ester	0.82	-	-
Acetic acid, trichloro-, nonyl ester	0.47	-	-
Nonyl chloroformate	0.32	-	-
**Others**			
2,4-Di-tert-butylphenol	3.66 ^a^	1.58 ^b^	1.55 ^b^
1,3-Dioxolane,4-methyl-2-pentyl-	-	0.45 ^b^	0.59 ^a^
(E)-2-Dodecene	-	0.26 ^b^	0.48 ^a^
Naphthalene	-	0.62 ^a^	0.33 ^b^
Indole	0.81 ^a^	0.30 ^b^	-
Azulene	1.01	-	-

FRN, WFRN, and WFRN-SPI represent the flat rice noodles, whole-grain flat rice noodles, and whole-grain flat rice noodles with soy protein isolate, respectively; compounds identified via GC-MS analysis are based on the comparison with retention indices (RI) and the mass spectra of the standard compounds (similarity ≥ 85%); relative content, the percentage of each compound area to total area of identified compounds; “-”, not detected; results are presented as means (*n* = 3). Means with different small letter superscripts in the same row are significantly different at *p* < 0.05.

## Data Availability

The data presented in this study are available on request from the corresponding author.
